# Supplementary material for: An integrated framework for quantifying immune-tumour interactions in a 3D co-culture model
Source: Commun Biol. 2021 Jun 24;4:781. doi: 10.1038/s42003-021-02296-7 (PMC8225809; doi:10.1038/s42003-021-02296-7)
Supplement: Supplementary file 2 — Supplementary Information [file 42003_2021_2296_MOESM2_ESM.pdf]

Supplementary Figure 1

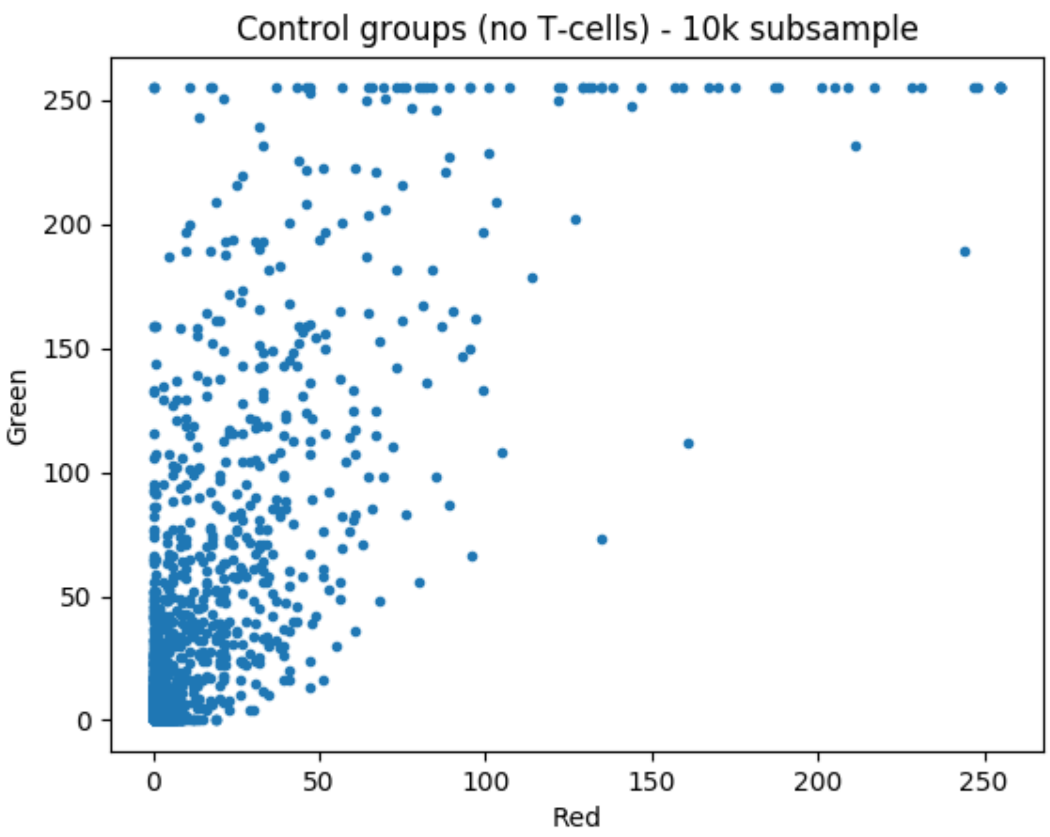

Supplementary Figure 1: Scatter plot of the red and green intensity value of pixels in the images of the control group (no immune cells). A random subsample of 10,000 pixels is shown, approximately 0.001% of the total dataset. A large amount of red channel intensity is evident despite the absence of immune cells and it appears there is a positive correlation between green channel intensity and red channel intensity which complicates the design of trafficking measures.

Supplementary Figure 2

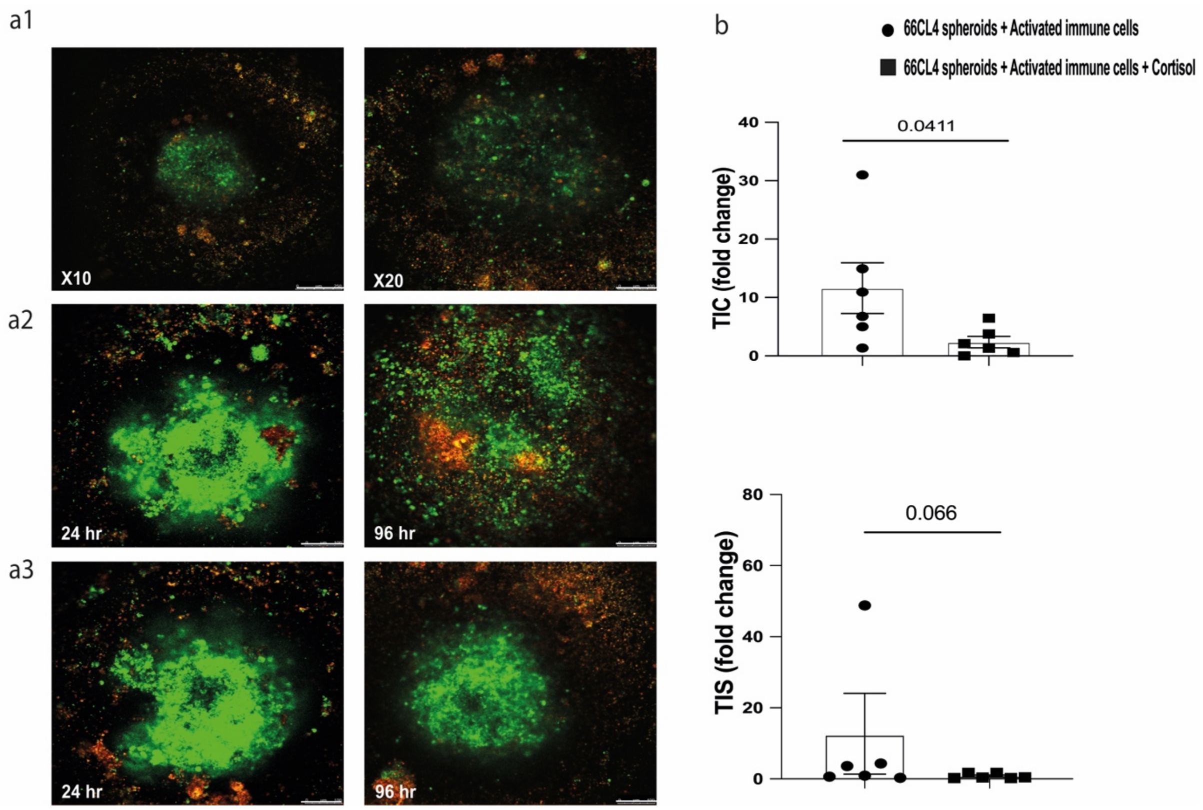

Supplementary Figure 2: High magnification images of the co-culture between 66CL4 spheroids and immune cells ± cortisol. A1. Same image of the co-culture at 10x vs at 20x. A2. 66CL4 spheroids + immune cells at 24 Vs 96 hr. A3. 66CL4 spheroids + immune cells + cortisol at 24 Vs 96 hr. B. The difference in infiltration ± cortisol presented as fold change using both TIS and TIC method. Statistical significance was calculated using a one-way ANOVA n=6 data represent mean ± SEM. * = p<0.05, ** = p<0.01, *** = p<0.001

Supplementary Figure 3

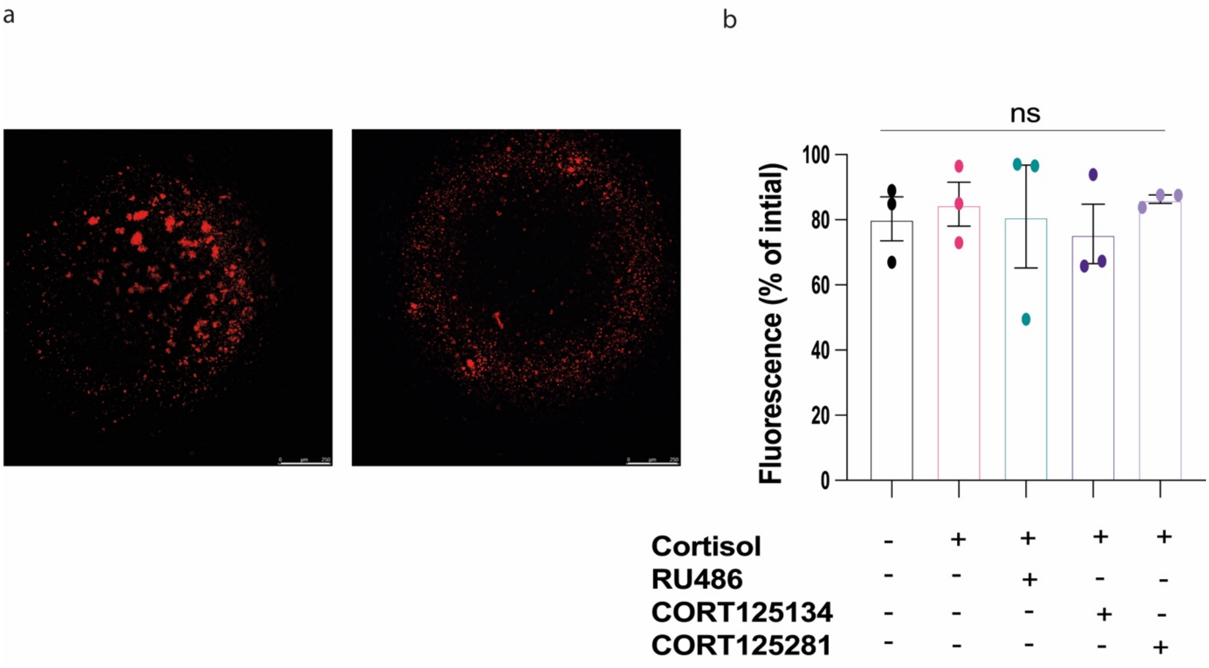

Supplementary Figure 3: The treatments used do not show a significant effect on the retention of splenocytes.
A. Representative images of splenocytes stained with the lipophilic tracer Dil D7777 at 0 hr and 96 hrs. The scale bar represents 250 µm B. No significant fluorescence of the splenocytes presented as a percentage of the initial fluorescence with the different treatments. Statistical significance was calculated using a one-way ANOVA n=3 data represent mean ± SEM. * = p<0.05, ** = p<0.01, *** = p<0.001

Supplementary Figure 4

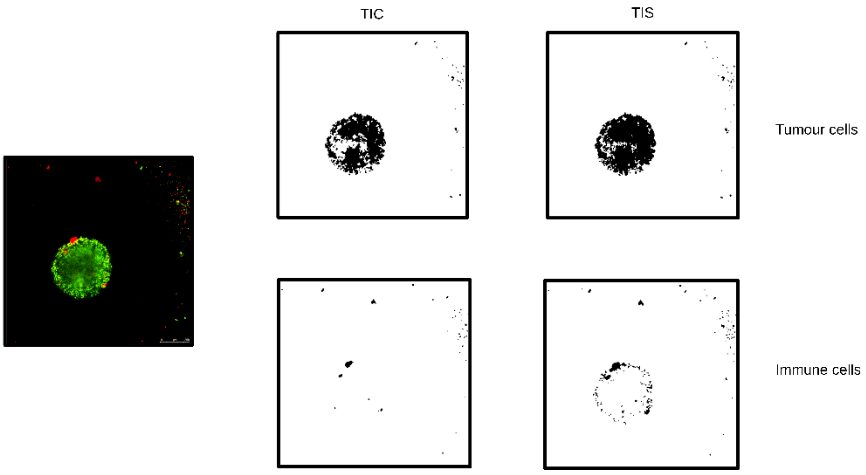

Supplementary Figure 4: Example of how the two different algorithms classify the image as tumour or immune cells. Left, the original image. Right, top row, regions classified as tumour cells by the algorithm underlying TIC (left column) and TIS (right column). Bottom row, regions classified as immune cells. The different underlying principles---a K-means classification for TIS, and a segmentation method for TIC---for each algorithm explain the differences in the regions identified as tumour or immune cells.

Supplementary Figure 5

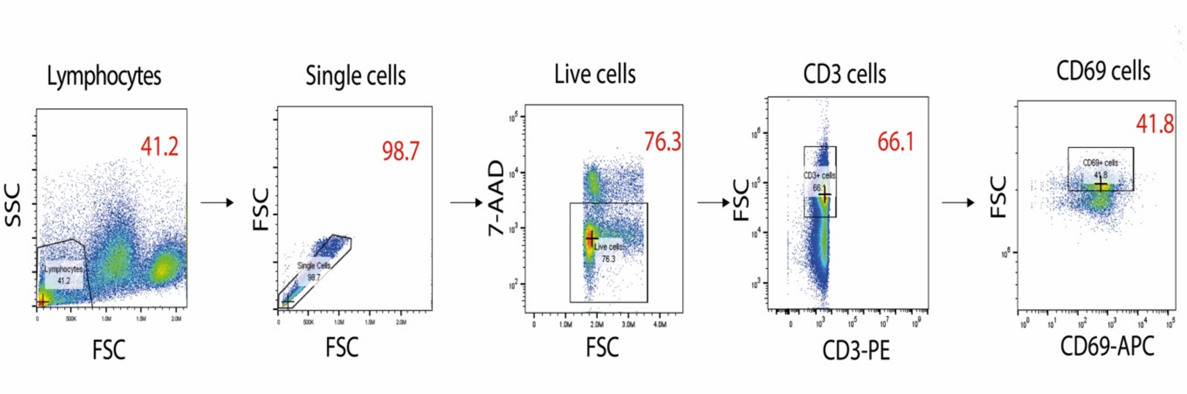

Supplementary Figure 5: Flow cytometry gating scheme for cells, lymphocytes, single cells, live cells, $CD3^+$ cells, and $CD69^+$ cells.
