# Supplementary material for: An integrated framework for quantifying immune-tumour interactions in a 3D co-culture model
Source: Commun Biol. 2021 Jun 24;4:781. doi: 10.1038/s42003-021-02296-7 (PMC8225809; doi:10.1038/s42003-021-02296-7)
Supplement: Supplementary file 3 — Reporting Summary [file 42003_2021_2296_MOESM3_ESM.pdf]

nature research

Corresponding author(s): Flint MS
Last updated by author(s): May 27, 2021

Reporting Summary

Nature Research wishes to improve the reproducibility of the work that we publish. This form provides structure for consistency and transparency in reporting. For further information on Nature Research policies, see our Editorial Policies and the Editorial Policy Checklist.

Statistics

For all statistical analyses, confirm that the following items are present in the figure legend, table legend, main text, or Methods section.

n/a	Confirmed	
☐	☒	The exact sample size (*n*) for each experimental group/condition, given as a discrete number and unit of measurement
☐	☒	A statement on whether measurements were taken from distinct samples or whether the same sample was measured repeatedly
☐	☒	The statistical test(s) used AND whether they are one- or two-sided<br>*Only common tests should be described solely by name; describe more complex techniques in the Methods section.*
☐	☒	A description of all covariates tested
☐	☒	A description of any assumptions or corrections, such as tests of normality and adjustment for multiple comparisons
☐	☒	A full description of the statistical parameters including central tendency (e.g. means) or other basic estimates (e.g. regression coefficient) AND variation (e.g. standard deviation) or associated estimates of uncertainty (e.g. confidence intervals)
☐	☒	For null hypothesis testing, the test statistic (e.g. *F*, *t*, *r*) with confidence intervals, effect sizes, degrees of freedom and *P* value noted<br>*Give P values as exact values whenever suitable.*
☒	☐	For Bayesian analysis, information on the choice of priors and Markov chain Monte Carlo settings
☒	☐	For hierarchical and complex designs, identification of the appropriate level for tests and full reporting of outcomes
☒	☐	Estimates of effect sizes (e.g. Cohen's *d*, Pearson's *r*), indicating how they were calculated

Our web collection on statistics for biologists contains articles on many of the points above.

Software and code

Policy information about availability of computer code

Data collection	images were acquired using the Zeiss software.
Data analysis	Python and Matlab were used analyse images, Here is the link to the Github repository:<br>https://github.com/ecam85/spheroids

For manuscripts utilizing custom algorithms or software that are central to the research but not yet described in published literature, software must be made available to editors and reviewers. We strongly encourage code deposition in a community repository (e.g. GitHub). See the Nature Research guidelines for submitting code & software for further information.

Data

Policy information about availability of data

All manuscripts must include a data availability statement. This statement should provide the following information, where applicable:
- Accession codes, unique identifiers, or web links for publicly available datasets
- A list of figures that have associated raw data
- A description of any restrictions on data availability

all raw data associated with the figures are available

Field-specific reporting

Please select the one below that is the best fit for your research. If you are not sure, read the appropriate sections before making your selection.

- [x] Life sciences
- [ ] Behavioural & social sciences
- [ ] Ecological, evolutionary & environmental sciences

For a reference copy of the document with all sections, see nature.com/documents/nr-reporting-summary-flat.pdf

Life sciences study design

All studies must disclose on these points even when the disclosure is negative.

Sample size	For all in vitro studies, experiments were carried as n = 6 unless otherwise specified. For the Elisa's 3 biological replicates and 2 technical replicates, for the flow we used (n =3 spleens) and 8 technical replicates. For in vivo experiments - estimates from preliminary data, n = 5 mice with two two stress groups (stress and non-stress) were used to determine the required sample size to reject the null hypothesis of no effect using methods for linear mixed models.
Data exclusions	N/A
Replication	Reproducibility was confirmed using a least 2 independent experiments for all in vitro experiments.
Randomization	Mice were implanted with tumour cells on the same day. Mice were randomly assigned experimental groups once the tumour volume reached 150-200mm3, which occurred within 1 week.
Blinding	Scoring/ image analysis of immunofluorescence images was performed blinded and analysis of CD3 in mouse tissue was performed blinded by another investigator.

Reporting for specific materials, systems and methods

We require information from authors about some types of materials, experimental systems and methods used in many studies. Here, indicate whether each material, system or method listed is relevant to your study. If you are not sure if a list item applies to your research, read the appropriate section before selecting a response.

Materials & experimental systems

n/a	Involved in the study	
	x	Antibodies
	x	Eukaryotic cell lines
x		Palaeontology and archaeology
	x	Animals and other organisms
x		Human research participants
x		Clinical data
x		Dual use research of concern

Methods

n/a	Involved in the study	
x		ChIP-seq
	x	Flow cytometry
x		MRI-based neuroimaging

Antibodies

Antibodies used	the murine PE-labelled anti-CD3 antibody at a concentration of 0.5 mg/ml and the APC-labelled anti-CD69 antibody at a concentration of 0.2 mg/ml (Biolegend, UK),
Validation	Both primary antibodies were validated by a titration experiment to choose the optimal working concentration.<br>Relevant citations for the CD69 antibody<br>Xie X, et al. 2009. J Immunol. 182:7163. PubMed<br>Xu L, et al. 2012. J Immunol. 188:248. PubMed<br>Lo W, Allen D 2012. Nat Immunol. 13:880. PubMed<br>Lin Y, et al. 2012. Cancer Prev Res. 5:1090. PubMed<br>Stepanek O, et al. 2013. J Immunol. 190:1807. PubMed<br>Michelet X, et al. 2015. J Immunol. 194:2079. PubMed<br>Morrison V, et al. 2015. J Immunol. 195: 105 - 115. PubMed<br>Olguín J, et al. 2015. Microbes Infect. 17: 586-595. PubMed<br>Schaffert S, et al. 2015. J Immunol. 195: 1470-1479. PubMed<br>Lebrun A, et al. 2015. J Immunol. 195: 4358 - 4368. PubMed<br>Hrdinka M, et al. 2016. PLoS One. 11: 0162863. PubMed<br>Woyciechowski S, Hofmann M, Pircher H 2017. Eur J Immunol. 47:244-250. PubMed<br>Relevant citations for the CD3 antibody

Vitoriano-Souza J, et al. 2012. PLoS One. 7:e40745. PubMed
Guo Y, et al. 2013. Infect Immun . 81:3923. PubMed
Gaudreault N, et al. 2013. Am J Pathol. 2:605. PubMed
MacDonald K, et al. 2014. J Immunol. 192:3180. PubMed
Motozono C, et al. 2014. Microbes Infect. 16:320. PubMed
Bergquist M, et al. 2014. J Infect. 69:113. PubMed
Martin R, et al. 2014. PLoS One. 9:110609. PubMed
Boulay A, et al. 2015. J Neurosci. 35:4427. PubMed
Kim P, et al. 2015. Infect Immun . 83: 2992 - 3002. PubMed
Vazquez S, et al. 2015. Exp Hematol. 43: 578-585. PubMed
Ma J, et al. 2016. Proc Natl Acad Sci U S A. 113: 450 - 458. PubMed
Kuo P, et al. 2016. J Am Heart Assoc. 5: 002610. PubMed
Campisi L, et al. 2016. Nat Immunol. 10.1038/ni.3512. PubMed
Qiu Z, et al. 2016. Sci Rep. 6:31936. PubMed
Zhang B, et al. 2016. Mol Cell. 63: 976-89. PubMed
D Kim, S Lee, H Jun 2016. Immunol Cell Biol. 10.1038/icb.2016.98. PubMed
Bansal K, et al. 2017. Nat Immunol. 18:263-273. PubMed
Cecchinato V, et al. 2017. J Immunol. 198(1):184-195. PubMed
Guo H, Cooper S, Friedman A, et al. 2017. PLoS One. 10.1371/journal.pone.0150809. PubMed
Ji XJ, et al. 2019. Mediators Inflamm. 2019:2750528. PubMed
Celià-Terrassa T, et al. 2017. Nat Cell Biol. 19:711. PubMed
LaFleur MW, et al. 2019. Nat Commun. 10:1668. PubMed
Kubli SP, et al. 2019. Nat Commun. 10:2678. PubMed
Logan K Smith et al. 2018. Immunity. 48(2):299-312 . PubMed
Malik A et al. 2018. Immunity. 49(3):515-530 . PubMed
Dyer DP et al. 2019. Immunity. 50(2):378-389 . PubMed
Wu J et al. 2017. Immunity. 47(6):1114-1128 . PubMed
Celis-Gutierrez J et al. 2019. Cell Rep. 27(11):3315-3330 . PubMed
Dave K et al. 2017. eLife. 6 pii: e23382. PubMed
Pan W et al. 2017. Immunity. 47(2):284-297 . PubMed
Qi S et al. 2016. eLife. 5 pii: e14756. PubMed
Kim I et al. 2015. Brain and behavior. 5(12):e00403 . PubMed
Sierro F, et al. 2017. Immunity. 47:374. PubMed
Leal AS, et al. 2019. Sci Rep. 5.286111111. PubMed
He L, et al. 2019. iScience. 0.790972222. PubMed
Kretschmer L, et al. 2020. Nat Commun. 0.536805556. PubMed
Jayachandran R, et al. 2019. Immunity. 50:152. PubMed
Qi S, et al. 2020. Theranostics. 10:1814. PubMed
Yu X, et al. 2020. Nat Commun. 11:1110. PubMed
Thelin MA, et al. 2017. Diabetes. 66:2220. PubMed
Grødeland G, et al. 2020. Front Immunol. 11:431. PubMed
Hiraishi Y, et al. 2018. Sci Rep. 8:18052. PubMed
LaFleur MW, et al. 2019. Nat Immunol. 20:1335. PubMed
Sarapulov AV, et al. 2020. Front Immunol. 11:599. PubMed
Davidson S, et al. 2020. Cell Rep. 31:107628. PubMed

Eukaryotic cell lines

Policy information about cell lines

Cell line source(s)	The 66CL4 cell line was kindly provided by Dr Erica Sloane (Monash University, Australia), and 4T1 cells from ATCC
Authentication	4T1 cells were purchased directly from ATCC, where they were authenticated. Cells were used within 10-15 passages of purchase. The mouse cell line 66CL4 was not authenticated as STR profiling is currently unavailable, see - A resource for cell line authentication, annotation and quality control -"Profiling still remains a challenge, as many mouse cell lines are derived from a handful of inbred strains and thus are indistinguishable, although SNP arrays may be able to resolve this problem. However, the chance of detecting mouse intra-species cross-contamination is low and development of a reliable test is needed." - Yu et al, Nature (2015)
Mycoplasma contamination	All cell lines tested negative for mycoplasma by PI taining
Commonly misidentified lines (See ICLAC register)	None

Animals and other organisms

Policy information about studies involving animals; ARRIVE guidelines recommended for reporting animal research

Laboratory animals	10-12-week-old, female BALB/c mice, weighing 18±2g, were purchased from Charles River laboratories (Margate, Kent, United Kingdom).

Wild animals	N/A
Field-collected samples	N/A
Ethics oversight	All in vivo studies were carried out with Home Office approval and approved by the Animal Welfare and Ethical Review Body (AWERB) at the University of Brighton

Note that full information on the approval of the study protocol must also be provided in the manuscript.

Flow Cytometry

Plots

Confirm that:

- [x] The axis labels state the marker and fluorochrome used (e.g. CD4-FITC).
- [x] The axis scales are clearly visible. Include numbers along axes only for bottom left plot of group (a 'group' is an analysis of identical markers).
- [x] All plots are contour plots with outliers or pseudocolor plots.
- [x] A numerical value for number of cells or percentage (with statistics) is provided.

Methodology

Sample preparation	The source of the cells is BALB/c mice spleens, mashed through a strainer, washed through RBCs lysis buffer and suspended in RPMI and 10% FBS
Instrument	The instrument used to acquire data is the BD Accuri C6
Software	The software used to do the analysis is Flowjo
Cell population abundance	Each spleen provides around 10^8 cells, tested using trypan blue followed by a 7AAD viability dye to show that cells are viable
Gating strategy	The gating was first done on unstained cells, followed by live cells and then either CD3 or CD69 positive cells

- [x] Tick this box to confirm that a figure exemplifying the gating strategy is provided in the Supplementary Information.
